# Natural antibacterial agents from arid-region pretreated lignocellulosic biomasses and extracts for the control of lactic acid bacteria in yeast fermentation

**DOI:** 10.1186/s13568-018-0654-8

**Published:** 2018-08-06

**Authors:** Sabeera Haris, Chuanji Fang, Juan-Rodrigo Bastidas-Oyanedel, Kristala Jones Prather, Jens Ejbye Schmidt, Mette Hedegaard Thomsen

**Affiliations:** 1 Department of Chemical Engineering, Khalifa University of Science and Technology, Masdar City Campus, P.O. Box 54224, Abu Dhabi, United Arab Emirates; 2 Department of Civil and Environmental Engineering, College of Engineering, United Arab Emirates University, P.O. Box 1755, Al-Ain, United Arab Emirates; 3 Department of Chemical Engineering, Massachusetts Institute of Technology, 77 Massachusetts Avenue, Cambridge, MA 02139 USA; 4 Department of Energy Technology, Aalborg University-Esbjerg Campus, Niels Bohrsvej 8, 6700 Esbjerg, Denmark; 5 Chemistry Department, Khalifa University of Science and Technology, Masdar City Campus, P.O. Box 54224, Abu Dhabi, United Arab Emirates

**Keywords:** Bacterial contamination, Lactic acid bacteria, Lignocellulosic biomass, Natural antibacterial, Yeast fermentation

## Abstract

Bacterial contamination is one of the major challenges faced by yeast fermentation industries as the contaminating microorganisms produce lactic acid and acetic acid, which reduces the viability of yeast, and hence fermentation yields. The primary bacterial contaminants of yeast fermentations are lactic acid bacteria (LAB). This study aims to identify potential natural antibacterial fractions from raw and pretreated lignocellulosic biomasses found in Abu Dhabi, UAE, in terms of LAB inhibition capacity, allowing growth of the yeast. The analysis was carried out using plating technique. Pretreatment liquid of the mangrove stem *Avicennia marina* hydrothermally pretreated at 210 °C exhibited the widest inhibition zone with an average diameter of 14.5 mm, followed by the pretreatment liquid of mangrove leaf pretreated at 190 °C, *Salicornia bigelovii* pretreated at 202 °C and rachis of date palm *Phoenix dactylifera* pretreated at 200 °C. The compounds responsible for the antibacterial activity will be characterized in further study.

## Introduction

Bacterial contamination is of significant concern for industrial yeast fermentation (Bischoff et al. 2007) as it decreases the available levels of carbohydrate and nutrients (Muthaiyan et al. 2011; Nwobi et al. 2015), leading to lower product yields and production of unwanted organic acids that may inhibit yeast growth, e.g. lactic acid (De Oliva-Neto and Yokoya 1998; Makanjuola et al. 1992; Leja and Broda 2009; Muthaiyan et al. 2011). Among the bacterial contaminants in industrial yeast fermentation, lactic acid bacteria (LAB) are the most prominent (Essia Ngang et al. 1990). The major LAB species, responsible for contamination, are *Lactobacillus* spp., *Leuconostoc* spp., and *Lactococci* spp. (Schell et al. 2007).

Conventionally in yeast fermentation, non-biological inhibitory agents have been implemented as contamination control agents. Antiseptics such as hydrogen peroxide, potassium metabisulfite (Chang et al. 1997), and 3,4,4-trichlorocarbanilide (De Oliva-Neto and Yokoya, 1998) have been shown to inhibit and control bacterial contamination in ethanol fermentations on a laboratory scale. However, full scale and pilot bioethanol plants employ acid washing and use antibiotics on a large scale to prevent contamination by lactic acid bacteria (De Oliva-Neto and Yokoya, 1998). The most common antibiotics employed are penicillin G, streptomycin, tetracycline (Bayrock et al. 2003), virginiamycin (Hynes et al. 1997) or mixtures of these antibiotics (Muthaiyan et al. 2011). Penicillin is added over a concentration of 1.5 mg/l, whereas virginiamycin is added in a range from 0.5 to 64 mg/l depending on the type of contaminant and tetracycline with a concentration ranging from 1 to 30 mg/l of which 60% gets retained in the fermentation mash (Muthaiyan et al. 2011). The use of antibiotics has however been challenged since their over-use has led to the creation of multi-resistant bacteria in both humans and animals (WHO 2001). Murphree et al. (2014) has reported the resistance of 32 LAB isolates to antibiotics, from eight different US bioethanol facilities, revealing the emergence of antibiotic resistant bacteria not only in medical field but also in fuel ethanol industries. With the widespread antibiotic resistance in fermentation plants and the possible public health threats, implementation of alternative contamination control methods and development of novel inhibitory antimicrobials are becoming essential (Muthaiyan et al. 2011).

Acid washing using sulfuric acid were used in breweries and fuel ethanol fermentation widely in-spite of the safety risk and the negative effect of acid on yeast viability (Ceccato-Antonini 2018). Chlorine dioxide; a strong oxidant with antimicrobial activity against bacteria viruses and spores; patented and commercialized as DuPont™ Fermasure^®^ has replaced antibiotics and 40% of acid washing in Brazilian distilleries (Ceccato-Antonini 2018). Controlling bacteria with other microorganisms has become a new strategy to curb contamination. Khatibi et al. (2014) demonstrated the reduction of *lactobacillus* contamination by employing yeast expressing a bacteriophage lytic enzyme (endolysins) during fuel ethanol fermentation from corn mash. On a small-scale, bacteriophages have been used to control LAB contamination in fermentation of corn mash (Liu et al. 2015). Rich et al. (2018) identified a group of harmless, beneficial strains of lactic acid bacteria that restored ethanol production to near normal levels when tested in combination with strains that cause stuck fermentations. Bacteriocins, which are antibacterial proteins produced by bacteria, such as niacin (Silva et al. 2014), nisin (Peng et al. 2012) have been shown to inhibit contaminant bacteria with no effect on yeast viability and fermentation. Since addition of purified bacteriocins in beer fermentation have been regulated by Germany purity law and governmental agencies (Ahn et al. 2017), studies involving plant derived antimicrobials are gaining interest.

Various natural compounds, plant derived compounds and extracts have been tested for contamination control in breweries (Muthaiyan et al. 2011). Gil et al. (2004) studied the antimicrobial activity of chitosan on beer spoilage bacteria and brewing yeasts and found that four strains of LAB and *Pediococcus* spp. could be inhibited in the presence of chitosan without affecting the viability of yeast. Flowers of hop plant are also used as substitutes to commercial antibiotics (Muthaiyan et al. 2011). Ruckle and Senn (2006) showed the potential use of hop acids as natural antibacterial preventing the production of lactic acid in distillery mashes for alcoholic fermentation.

In this study, hydrothermal pretreatment liquids (liquors) and water extracts of various plant biomasses native to arid areas were tested for their antibacterial activity against lactic acid bacteria. The plant biomass used for the study were parts of date palm *Phoenix dactlylifera*—leaflets and rachis; landscaping clippings, *Cynodon dactylon* (Bermuda grass) and *Clerodendrum inerme* (Jasmine Hedges); the terrestrial halophyte species *Salicornia bigelovii*, *Salicornia sinus persica* and *Suaeda iranshahari*; and the mangrove *Avicennia marina*.

## Materials and methods

For the antibacterial analysis, the samples used were the hydrothermal pretreatment liquid of various plant biomasses and water extracts of certain plant biomass which are as follows: date palm leaflets Pretreated at 200 °C; date palm leaflets pretreated in seawater at 200 and 210 °C; date palm rachis pretreated at 180, 190, 200 and 210 °C; mangrove stem *A. marina* pretreated at 190, 200 and 210 °C; mangrove leaf *A. marina* pretreated at 190, 200 and 210 °C; *S. bigelovii* pretreated at 118,160 and 200 °C; *A. marina* stem water extract; *S. sinus persicus* water extract; halophyte stem *S. iranshahari* water extract; halophyte Leaf *S. iranshahari* water extract; Jasmine Hedges water extract; and Bermuda grass water extract. Based on the lignocellulosic composition (Ashraf et al. 2016), plant such as date palm, mangrove and *S. bigelovii* which had high glucan and xylan content were subjected to hydrothermal pretreatment or liquid hot water treatment (Hendricks and Zeeman 2009) and halophytes and landscaping clippings were subjected to Soxhlet water extraction, for the digestion of lignocellulosic structure. The pretreatment liquids used for the study are listed in Table 1 and the water extracts are listed in Table 2. The temperature profile was selected based on severity factor: low, medium and high temperature for low (1.83), medium (3.07) and high (4.3) severity factors. Different severity factors have different digestibility effects on the lignocellulosic biomass, resulting in releasing of different chemical compounds which has inhibitory and antibacterial properties (Hendricks and Zeeman 2009). So different temperatures where selected to find out which severity factor had the better antibacterial effect against LAB. All plants used in the study were collected from nearby locality, the details of which are given by Ashraf et al. (2016).

**Table 1:** The pretreated liquid of various plant biomasses along with their pretreatment conditions

Plant type	Pretreatment conditions
Date palm leaflets	200 °C
Date palm leaflets	200 and 210 °C in seawater
Date palm rachis	180, 190, 200 and 210 °C
Mangrove stem *A.marina*	190, 200 and 210 °C
Mangrove leaf *A.marina*	190, 200 and 210 °C
*S.bigelovii*	118,160 and 202 °C

**Table 2:** Water extracts of the plants used

Plant	Plant part
*A.marina*	Stem
*S.sinus persica*	Whole plant
*S.iranshahari*	Stem
*S.iranshahari*	Leaf
Jasmine Hedges	Whole plant
Bermuda grass	Whole plant

### Preparation of pretreated liquid of biomass

Each fraction of the plant (Table 1) was cut and milled to around 1 mm. The milled biomass was subjected to hydrothermal pretreatment in a Parr reactor (Parr Instrument Company, Moline, Illinois) at various temperatures (118 °C, 160 °C, 190 °C, 200 °C and 210 °C) for 10 min with dry matter loading of 10% in distilled water (Fang et al. 2015). After pretreatment, the slurry was filtered through a vacuum filter to separate the filtrate (liquid fraction) and filter cake (fiber fraction). The liquid fraction known as pretreated liquid was used in the antibacterial assay. The pretreatment with seawater was performed in the similar way except that seawater was used instead of distilled water. Pretreated liquid from seawater was used for the study to see if these exhibited antibacterial property similar to those from freshwater. Pretreatment with seawater could help reduce the stress on the freshwater use, which is a limited resource in arid areas (Bastidas-Oyanedel, et al. 2016).

### Preparation of water extracts of biomass

To prepare the water extract of the biomass, 5 g of dry biomass was transferred to a pre-weighed, tarred and dry cellulose thimble which was placed in a Soxhlet extractor connected to a collection flask containing 200 ml distilled water. An Allihn condenser was fitted on the boiling flask and cool water was run through the condenser. Upon completion in 10 h, the water from the extractor body was collected in a flask (Sluiter et al. 2008). The water extract was weighed and stored in the freezer until use in the antibacterial analysis and inhibition zone analysis.

### Microorganism

The microorganisms used in this study were *Lactococcus lactis* and *Saccharomyces cerevisae* (yeast). Pure strains of yeast was isolated from commercially available Baker’s yeast (brand name: Torrjast Instant yeast, COOP Trading A/S, Frankfurt, Germany) and LAB was isolated from cheese culture (brand name: Danisco cheese starter culture HMM4, Niebüll, Germany). Genetically modified strain of *E. coli* and *S. cerevisiae* were also used for this study, to see if the extracts under study could be used for contamination control in fermentations involving the genetically modified strains. The *S. cerevisiae* strain (*CEN.PK2*-*1D*) was engineered for the production of glucaric acid which has therapeutic values and has been classified as a top 12 value added product from biomass by the US. Department of Energy (Gupta et al. 2016). The recombinant *E. coli* strain was developed for the biosynthesis of 3-hydroxy-γ-butyrolactone and 3,4-dihydroxybutyricacid, which is a precursor in the synthesis of a variety of pharmaceuticals, solvents and polymers (Dhamankar et al. 2014).

### Preparation of growth media

The LAB culture was grown on MRS (de Mann Rogosa Sharpe) medium and the yeast was grown on YPD (Yeast extract, Peptone, Dextrose) medium. The *E. coli* were grown on LB (Lysogeny broth) medium as described by Dhamankar et al. (2014). The MRS medium composition was 1% peptone, 0.8% egg extract, 0.4% yeast extract, 2% d-glucose, 0.5% sodium acetate trihydrate, 0.1% polysorbate 80 (Tween 80), 0.2% dipotassium hydrogen phosphate, 0.2% triammonium citrate, 0.02% magnesium sulfate heptahydrate, and 0.005% manganese sulfate tetrahydrate. The YPD medium was composed of 1% yeast extract, 2% peptone, and 2% d-glucose. The agar plates were prepared by adding 1.5% agar to the media and pH adjusted to 6.5 ± 0.2 at 25 °C. All the media were autoclaved before inoculation.

### Antibacterial analysis of the pretreated liquids and water extracts

1 ml of each of the sample was transferred to autoclaved culture tubes. 10% LAB in MRS medium was inoculated into one set of tubes containing the sample. Similarly yeast in YPD medium was inoculated into the second set of tubes with the sample. The third set of samples was inoculated with *E. coli*. Tubes with LAB were incubated at 40 °C whereas the tubes with yeast and *E. coli* were incubated at 30 °C, both for 48 h. The samples with LAB, yeast and *E. coli* were spread on MRS agar petri dishes, YPD agar petridish and LB agar petridishes, respectively. MRS agar petridishes were incubated at 40 °C and the YPD and LB agar petridishes were incubated at 30 °C. The growth of the microorganisms was observed for 5 days. Control petridishes were also prepared. Positive control petridish were MRS agar with LAB; YPD agar with yeast and LB agar with *E. coli*. Negative control plates were those with media alone, without any microorganisms or sample.

### Inhibition Zone Test

The Inhibition Zone Test, also called a Kirby-Bauer disk diffusion test (Fleming and Etchells 1967), was performed as a second test to evaluate the effect of selected pretreatment liquids and water extracts on the growth of *L.lactis*. The assays were carried out in disposable petri dishes with MRS agar medium. The disks used were of size of 6 mm diameter from the Whatman filter paper (No.5). Bacterial inhibition zones were determined for the pretreated liquids and extracts of various biomasses that were identified in the antibacterial analysis to be potentially antimicrobial, inhibiting LAB but not yeast or *E. coli*. 50 uL of the LAB was inoculated from an overnight culture onto each petri-dish and was spread on the surface of the agar using a sterile L-loop. Each disk was dipped into each potential antimicrobial sample. The excess liquid was drained by touching onto the sides of the tube with the sample. The disks were then placed onto the agar plates using sterile forceps. Disks dipped into the 50% diluted form of the same antimicrobial samples were also placed on the petri dish. Each petri dish contained duplicates of the sample. The petri dishes were incubated at 40 °C for 5 days. The size of the inhibition zone was measured at the widest point.

## Results

### Antibacterial analysis of the biomass extracts and pretreated liquids

Among the 22 samples analyzed, 10 of them showed the desired characteristic of inhibiting the growth of LAB and not that of the yeast or *E. coli*. The growth of microorganisms was observed and listed as in Table 3. The growth of all microorganisms was observed when pretreatment liquid of date palm leaflets at 200 °C, *S.bigelovii* at 118 and 160 °C, and the water extracts of *A.marina* Stem, halophyte stem *S.iranshahari*, halophyte leaf *S.iranshahari*, Jasmine Hedges, and Bermuda grass were added. This implies these pretreated liquids and water extracts have no inhibitory action against the microorganisms under study. No growth of LAB and yeast was observed when inoculated with pretreatment liquid of date palm rachis at 210 °C, *A.marina* stem at 190 °C, *A.marina* leaf treated at 200 and 210 °C, indicating that they contain compounds that are inhibitory to both LAB and yeast. The following extracts shows the desired characteristics of inhibiting LAB but not the yeast: date palm leaflets pretreated in seawater at 200 and 210 °C, date palm rachis pretreated at 180, 190 and 200 °C; mangrove stem *A.marina* pretreated at 200 and 210 C, mangrove leaf *A.marina* pretreated at 190 °C, *S.bigelovii* pretreated at 202 °C, and water extract of *S.sinus persica.* These samples could be used as potential antibacterial agents in fermentation industries where yeast is the driver and LAB the contaminant. Also, no growth inhibition was observed when the extracts were added to both the genetically modified strains. This again assures that these extracts can be used against LAB contaminants in the fermentation involving the *S. cerevisiae* strain (*CEN.PK2*-*1D*) and the recombinant *E. coli* strain. The growth in positive control petri dishes showed the viability of the microorganisms used and no growth in negative control denotes the sterility of the growth media used.

**Table 3:** Growth and inhibition in various biomass samples

Extracts	Growth			
	LAB	Yeast	E. coli*	Yeast*
Date palm leaflets-PT 200 °C	+	+	+	+
Date palm leaflets -PT 200 °C in seawater	−	+	+	+
Date palm leaflets-PT 210 °C in seawater	−	+	+	+
Date palm rachis-PT 180 °C	−	+	+	+
Date palm rachis-PT 190 °C	−	+	+	+
Date palm rachis-PT 200 °C	−	+	+	+
Date palm rachis-PT 210 °C	−	−	+	+
Mangrove stem *A.marin*-PT 190 °C	−	−	+	+
Mangrove stem *A.marina*-PT 200 °C	−	+	+	+
Mangrove stem *A.marina*-PT 210 °C	−	+	+	+
Mangrove leaf *A.marina*-PT 190 °C	−	+	+	+
Mangrove leaf *A.marina*-PT 200 °C	−	−	+	+
Mangrove leaf *A.marina*-PT 210 °C	−	−	+	+
*S.bigelovii*-PT 118 °C	+	+	+	+
*S.bigelovii*-PT 160 °C	+	+	+	+
*S.bigelovii* - PT 202 °C	−	+	+	+
*A.marina* stem- WE	+	+	+	+
*S.sinus persicus* –*WE*	−	+	+	+
Halophyte stem *S.iranshahari*-WE	+	+	+	+
Halophyte leaf *S.iranshahari*-WE	+	+	+	+
Jasmine hedges-WE	+	+	+	+
Bermuda grass–WE	+	+	+	+
Positive control	+	+	+	+
Negative control	−	−	−	−

+  growth of microorganism; −denotes no growth; *PT* pretreated, *WE* water extract; E. coli* and yeast* are the genetically modified strains

### Inhibition Zone Study

**Fig. 1:**
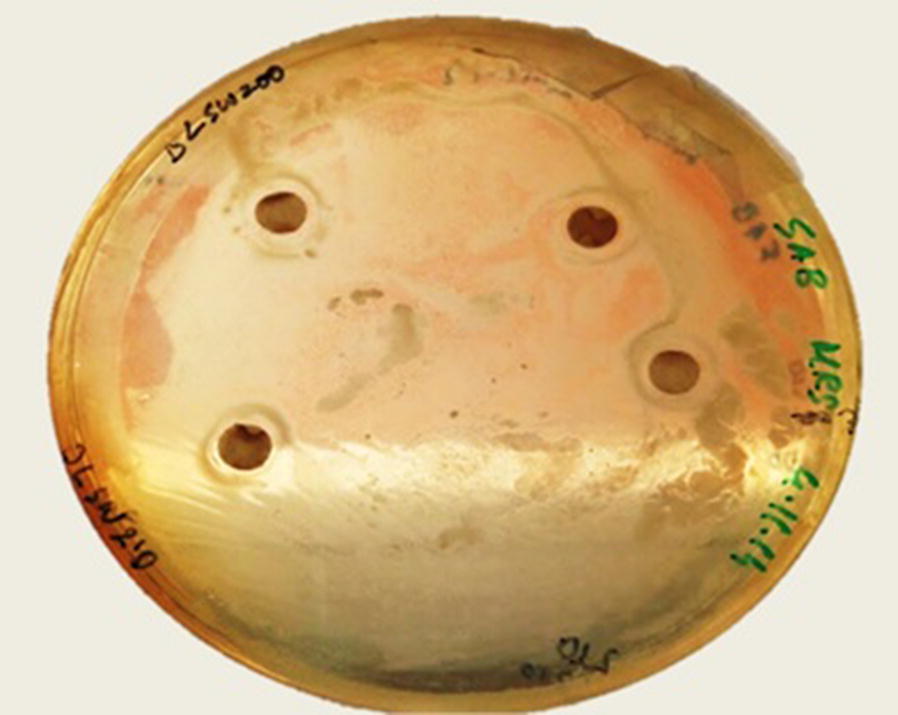
Inhibition zone for date palm leaflets PT 200 °C in seawater and date palm leaflets PT 210 °C in seawater

After 24 h of incubation, the selected samples (samples which inhibited the growth of LAB and not that of yeast) showed zone of inhibition around the disk as shown in Fig. 1. The diameter of the inhibition zones was measured at the widest point. The measure of zone diameter is proportional to the inhibitory action (Gaydos and Harrington 1982). The widest zone is responsible for the highest inhibition. The average zone diameters of the duplicates were tabulated in Table 4.

**Table 4:** Average zone diameter for various samples

Sample	Average zone diameter in mm	
	Undiluted	50% diluted
Date palm leaflets PT 200 °C in seawater	8 ± 0.01	No zone
Date palm leaflets PT 210 °C in seawater	6.5 ± 0.707	No zone
Date palm rachis-PT 180 °C	6.5 ± 0.707	No zone
Date palm rachis-PT 190 °C	6.5 ± 0.707	No zone
Date palm rachis-PT 200 °C	10 ± 0.01	No zone
Mangrove stem *A.marina*-PT 200 °C	9 ± 1.414	No zone
Mangrove stem *A.marina*-PT 210 °C	14.5 ± 0.707	11.5 ± 0.707
Mangrove leaf *A.marina*-PT 190 °C	12 ± 1.414	10.5 ± 0.707
*S.bigelovii*-PT 202 °C	11 ± 0.01	No zone
*S.sinus persica*-WE	8 ± 0.01	No zone

The experimental error was calculated as the standard deviation of duplicates

* PT* pretreated; *WE* water extract)

The widest inhibition zone was exhibited by the mangrove stem *A.marina* extract pretreated at 210 °C, with an average zone diameter of 14.5 mm with the undiluted form of pretreatment liquid and 11.5 mm with 50% diluted form. The pretreatment liquid of mangrove leaf *A.marina* at 190 °C exhibited an average zone diameter of 12 mm with undiluted form and 10.5 mm with 50% diluted form. The pretreatment liquid of *S.bigelovii* at 202 °C exhibited an average zone diameter of 11 mm and date palm rachis treated at 200 °C with 10 mm of average zone diameter. No zone of inhibition was observed for some the extracts at 50% dilution in Table 4, which could be because the pretreated liquid and water extract used in the study were not in the concentrated form. On further dilution, the antibacterial compound may be too weak to exhibit the inhibitory property. The active compound(s) responsible for the inhibitory action could be phenolic compounds formed due to lignin degradation, which may be identified using a Gas Chromatography-Mass Spectroscopy (GC–MS) (Proestos et al. 2006). The minimum inhibitory concentration also needs to be determined in further studies.

## Discussion

The pretreated liquids and extracts studied thus exhibited antibacterial properties towards the LAB, which is one of the major bacterial contaminant in the yeast fermentations. These extracts also showed not to inhibit the yeast growth, which makes the samples a potential candidate to replace the commercial antibiotics in yeast fermentation industries. These alternate antimicrobials could control the after effects of misusing the antibiotics in industries, such as emergence of antimicrobial resistant microbes to some extent. Antibacterial properties of *A.marina* has been proven against urinary tract infection bacterial pathogens by (Ravikumar et al. 2010) and against *Staphylococcus aureus* by (Dhayanithi et al. 2012); however, no study has been carried out against the LAB contaminants. Similarly, Al-Zoreky and Al-Taher (2015) demonstrated the antibacterial activity of the spathe of the date palm against food borne pathogens and Kchaou et al. (2016) studied the antibacterial property of second grade extracts of date palm against human pathogens. No antibacterial property of the halophytes *S.bigelovii* and *S.sinus persica* against LAB has been reported before. Other plant derived compounds which have proven to prevent contamination include essential oils. Neyret et al. (2014) has shown promising results on the effect of essential oils such as thymol, carvacrol, eugenol, trans-cinna-maldehyde and α-terpineol, to control the formation of biofilms. Gyawali and Ibrahim (2014) have reviewed many plant by-products such as fruit peels, fruit seeds, coconut husk and other compounds of animal and bacterial origin to have antibacterial property.

Potential antimicrobial agents from an arid-region lignocellulosic biomass were identified. The pretreatment liquid of the stem of the mangrove *A.marina* at 210 °C was identified to be the most inhibitory for lactic acid bacteria. These antimicrobials did not inhibit yeast and *E. coli*, but only LAB. The broad spectrum inhibitory action of these antimicrobials can be identified by performing the same analysis on different LAB strains. The active compound(s) and the minimum inhibitory concentration also need to be determined. With further studies, these identified antimicrobials could potentially be an alternate to the commercial antibiotics for contamination control in fermentation industries.

## Abbreviations

LAB: lactic acid bacteria
MRS: de Mann Rogosa Sharpe
YPD: yeast extract, peptone, dextrose
LB: Lysogeny broth

## References

[CR1] Ahn H, Kim J, Kim WJ (2017). Isolation and characterization of bacteriocin-producing *Pediococcus acidilactici* HW01 from malt and its potential to control beer spoilage lactic acid bacteria. Food Control.

[CR2] Al-Zoreky NS, Al-Taher AY (2015). Antibacterial activity of spathe from *Phoenix dactylifera L.* against some food-borne pathogens. Ind Crops Prod.

[CR3] Ashraf MT, Fang C, Bochenski T, Cybulska I, Alassali A (2016). Estimation of bioenergy potential for local biomass in the United Arab Emirates. EJFA.

[CR4] Bastidas-Oyanedel JR, Fang C, Almardeai S, Javid U, Yousuf A, Schmidt JE (2016). Waste biorefinery in arid/semi-arid regions. Bioresour Technol.

[CR5] Bayrock DP, Thomas KC, Ingledew WM (2003). Control of *Lactobacillus* contaminants in continuous fuel ethanol fermentations by constant or pulsed addition of penicillin G. Appl Microbiol Biotechnol.

[CR6] Bischoff KM, Skinner-Nemec KA, Leathers TD (2007). Antimicrobial susceptibility of *Lactobacillus* species isolated from commercial ethanol plants. J Ind Microbiol Biotechnol.

[CR7] Ceccato-Antonini SR (2018). Conventional and nonconventional strategies for controlling bacterial contamination in fuel ethanol fermentations. World J Microb Biotechnol.

[CR8] Chang IS, Kim BH, Shin PK (1997). Use of sulfite and hydrogen peroxide to control bacterial contamination in ethanol these include: use of sulfite and hydrogen peroxide to control bacterial contamination in ethanol fermentation. Appl Environ Microbiol.

[CR9] De Oliva-Neto P, Yokoya F (1998). Effect of 3,4,4′-trichlorocarbanilide on growth of lactic acid bacteria contaminants in alcoholic fermentation. Bioresour Technol.

[CR10] Dhamankar H, Tarasova Y, Martin CH, Prather KLJ (2014). Engineering *E. coli* for the biosynthesis of 3-hydroxy-butyrolactone (3HBL) and 3,4-dihydroxybutyric acid (3,4-DHBA) as value-added chemicals from glucose as a sole carbon source. Metab Eng.

[CR11] Dhayanithi NB, Kumar TTA, Murthy RG, Kathiresan K (2012). Isolation of antibacterials from the mangrove, *Avicennia marina* and their activity against multi drug resistant *Staphylococcus aureus*. Asian Pac J Trop Biomed.

[CR12] Essia Ngang JJ, Letourneau F, Wolniewicz E, Villa P (1990). Inhibition of beet molasses alcoholic fermentation by *lactobacilli*. Appl Microbiol Biotechnol.

[CR13] Fang C, Schmidt JE, Cybulska I, Brudecki GP, Frankær CG, Thomsen MH (2015). Hydrothermal pretreatment of date palm (*Phoenix dactylifera L*.) leaflets and rachis to enhance enzymatic digestibility and bioethanol potential. Biomed Res Int.

[CR14] Fleming HP, Etchells JL (1967). Occurrence of an inhibitor of lactic acid bacteria in green olives. Appl Microbiol.

[CR15] Gaydos JM, Harrington BJ (1982). Agar disk diffusion for the quality control testing of autobac elution disks. Antimicrob Agents Chemother.

[CR16] Gil G, Del Mónaco S, Cerrutti P, Galvagno M (2004). Selective antimicrobial activity of chitosan on beer spoilage bacteria and brewing yeasts. Biotechnol Lett.

[CR17] Gupta A, Hicks MA, Manchester SP, Prather KL (2016). Porting the synthetic d-glucaric acid pathway from *Escherichia coli* to *Saccharomyces cerevisiae*. Biotechnol J.

[CR18] Gyawali R, Ibrahim SA (2014). Natural products as antimicrobial agents. Food Control.

[CR19] Hendricks ATWM, Zeeman G (2009). Pretreatments to enhance the digestibility of lignocellulosic biomass. Bioresour Technol.

[CR20] Hynes SH, Kjarsgaard DM, Thomas KC, Ingledew WM (1997). Use of virginiamycin to control the growth of lactic acid bacteria during alcohol fermentation. J Ind Microbiol Biotechnol.

[CR21] Kchaou W, Abbès F, Mansour R, Blecker C, Attia H, Besbes S (2016). Phenolic profile, antibacterial and cytotoxic properties of second grade date extract from Tunisian cultivars (*Phoenix dactylifera L.*). Food Chem.

[CR22] Khatibi PA, Roach DR, Donovan DM, Hughes SR, Bischoff KM (2014). *Saccharomyces cerevisiae* expressing bacteriophage endolysins reduce Lactobacillus contamination during fermentation. Biotechnol Biofuels.

[CR23] Leja K, Broda M (2009). The occurrence and identification of microbiological contamination in fuel ethanol production. Acta Sci Pol Technol Aliment.

[CR24] Liu M, Bischoff KM, Gill JJ, Mire-Criscione MD, Berry JD, Young R, Summer EJ (2015). Bacteriophage application restores ethanol fermentation characteristics disrupted by *Lactobacillus fermentum*. Biotechnol Biofuels.

[CR25] Makanjuola DB, Tymon A, Springham DG (1992). Some effects of lactic acid bacteria on laboratory-scale yeast fermentations. Enzyme Microb Technol.

[CR26] Murphree CA, Heist EP, Moe LA (2014). Antibiotic resistance among cultured bacterial isolates from bioethanol fermentation facilities across the United States. Curr Microbiol.

[CR27] Muthaiyan A, Limayem A, Ricke SC (2011). Antimicrobial strategies for limiting bacterial contaminants in fuel bioethanol fermentations. Prog Energy Combust Sci.

[CR28] Neyret C, Herry JM, Meylheuc T, Dubois-Brissonnet F (2014). Plant-derived compounds as natural antimicrobials to control paper mill biofilms. J Ind Microbiol Biotechnol.

[CR29] Nwobi A, Cybulska I, Tesfai W, Shatilla Y, Rodríguez J, Thomsen MH (2015). Simultaneous saccharification and fermentation of solid household waste following mild pretreatment using a mix of hydrolytic enzymes in combination with *Saccharomyces cerevisiae*. Appl Microbiol Biotechnol.

[CR30] Peng J, Zhang L, Gu ZH, Ding ZY, Shi GY (2012). The role of nisin in fuel ethanol production with *Saccharomyces cerevisiae*. Lett Appl Microbiol.

[CR31] Proestos C, Sereli D, Komaitis M (2006). Determination of phenolic compounds in aromatic plants by RP-HPLC and GC-MS. Food Chem.

[CR32] Ravikumar S, Gnanadesigan M, Suganthi P, Ramalakshmi A (2010). Antibacterial potential of chosen mangrove plants against isolated urinary tract infectious bacterial pathogens. Int J Med Sci.

[CR33] Rich JO, Bischoff KM, Leathers TD, Anderson AM, Liu S, Skory CD (2018). Resolving bacterial contamination of fuel ethanol fermentations with beneficial bacteria—an alternative to antibiotic treatment. Bioresour Technol.

[CR34] Ruckle L, Senn T (2006). Hop acids can efficiently replace antibiotics in ethanol production. Int Sugar J.

[CR35] Schell DJ, Dowe N, Ibsen KN, Riley CJ, Ruth MF, Lumpkin RE (2007). Contaminant occurrence, identification and control in a pilot-scale corn fiber to ethanol conversion process. Bioresour Technol.

[CR36] Silva SS, Vitolo M, González JMD, Oliveira RPS (2014). Overview of *Lactobacillus plantarum* as a promising bacteriocin producer among lactic acid bacteria. Food Res Int.

[CR37] Sluiter A, Ruiz R, Scarlata C, Sluiter JA, Templeton D (2008) Determination of extractives in biomass: laboratory analytical procedure (LAP). Tech Rep. NREL/TP-510-42619 1–9

[CR38] WHO (2001) WHO Global strategy for containment of antimicrobial strategy for containment of antimicrobial resistance. World Health WHO/CDS/CS 105. WHO/CDS/CSR/DRS/2001.2. p. 99

